# Supplementary material for: Top predator reveals the stability of prey community in the western subarctic Pacific
Source: PLoS One. 2020 Jun 19;15(6):e0234905. doi: 10.1371/journal.pone.0234905 (PMC7304915; doi:10.1371/journal.pone.0234905)
Supplement: S1 Table — AIC, Akaike information criterion; BIC, Bayesian information criterion; DF, degree of freedom; logLik., maximum log-likelihood ratio; edf, estimated degrees of freedom; Ref.df, reference degree of freedom (prior to deductions); R-sq.(adj), adjusted R-squared; Std.Dev., standard deviation; Std.Error, standard error. (DOCX) [file pone.0234905.s001.docx]

**S1 Table** GAMM results for stable isotopes (δ^15^N and δ^13^C) for *Ommastrephes bartramii* modeled in relation to monthly mean sea surface temperature (SST) and chlorophyll a concentration (Chl-*a*) in the western Subarctic Gyre of northwest Pacific Ocean. AIC, Akaike information criterion; BIC, Bayesian information criterion; DF, degree of freedom; logLik., maximum log-likelihood ratio; edf, estimated degrees of freedom; Ref.df, reference degree of freedom (prior to deductions); R-sq.(adj), adjusted R-squared; Std.Dev., standard deviation; Std.Error, standard error.

(a) δ^15^N

| LME Random effects | Std.Dev. |  |  |  |
| --- | --- | --- | --- | --- |
| ~\|sampling month | 2.74E-07 |  |  |  |
| Residual | 0.95 |  |  |  |
| AIC | BIC | logLik |  |  |
| 227.05 | 238.89 | -108.52 |  |  |
| GAM | Estimate | Std. Error | t value | Pr(>\|t\|) |
| (Intercept) | 8.42 | 0.12 | 78 | <2e-16 |
|  | edf | Ref.df | F | p-value |
| s(Chl-*a*) | 3.55E-01 | 9 | 0.061 | 0.22 |
| s(SST) | 5.98E-08 | 9 | 0 | 0.92 |
| R-sq.(adj) | 0.0068 |  |  |  |

(b) δ^13^C

| LME Random effects | Std.Dev. |  |  |  |
| --- | --- | --- | --- | --- |
| ~\|sampling month | 4.54E-11 |  |  |  |
| Residual | 0.69 |  |  |  |
| AIC | BIC | logLik |  |  |
| 176.9898 | 188.84 | -83.49 |  |  |
| GAM | Estimate | Std. Error | t value | Pr(>\|t\|) |
| (Intercept) | -20.48 | 0.079 | -259.90 | <2e-16 |
|  | edf | Ref.df | F | p-value |
| s(Chl-*a*) | 7.96E-08 | 9 | 0 | 0.55 |
| s(SST) | 4.07E-08 | 9 | 0 | 0.67 |
| R-sq.(adj) | -5.88E-10 |  |  |  |
