# Supplementary material for: Top predator reveals the stability of prey community in the western subarctic Pacific
Source: PLoS One. 2020 Jun 19;15(6):e0234905. doi: 10.1371/journal.pone.0234905 (PMC7304915; doi:10.1371/journal.pone.0234905)
Supplement: S2 Table — The fatty acids used for GAMM are those differed significantly between sampling months (details see Table 1). Akaike information criterion; BIC, Bayesian information criterion; DF, degree of freedom; logLik., maximum log-likelihood ratio; edf, estimated degrees of freedom; Ref.df, reference degree of freedom (prior to deductions); R-sq.(adj), adjusted R-squared; Std.Dev., standard deviation; Std.Error, standard error. (DOCX) [file pone.0234905.s002.docx]

**S2 Table** GAMM results for fatty acids of *Ommastrephes bartramii* modeled in relation to monthly mean sea surface temperature (SST) and chlorophyll a concentration (Chl-*a*) in the western Subarctic Gyre of northwest Pacific Ocean. The fatty acids used for GAMM are those differed significantly between sampling months (details see Table 1). Akaike information criterion; BIC, Bayesian information criterion; DF, degree of freedom; logLik., maximum log-likelihood ratio; edf, estimated degrees of freedom; Ref.df, reference degree of freedom (prior to deductions); R-sq.(adj), adjusted R-squared; Std.Dev., standard deviation; Std.Error, standard error.

**(1) 11:0**

| LME Random effects | SD |  |  |  |
| --- | --- | --- | --- | --- |
| ~\|sampling month | 4.86E-18 |  |  |  |
| Residual | 0.07 |  |  |  |
| AIC | BIC | logLik |  |  |
| -294.47 | -280.17 | 152.24 |  |  |
| GAM | Estimate | Std. Error | t value | Pr(>\|t\|) |
| (Intercept) | 0.17 | 0.0066 | 25.18 | <2e-16 |
|  | edf | Ref.df | F | p-value |
| s(chla.mn) | 7.15E-08 | 9 | 0 | 0.83 |
| s(sst.mn) | 9.94E-08 | 9 | 0 | 0.39 |
| R-sq.(adj) | -1.31E-10 |  |  |  |

**(2) 13:0**

| LME Random effects | SD |  |  |  |
| --- | --- | --- | --- | --- |
| ~\|sampling month | 0.06 |  |  |  |
| Residual | 0.17 |  |  |  |
| AIC | BIC | logLik |  |  |
| -67.65 | -53.35 | 38.82 |  |  |
| GAM | Estimate | Std. Error | t value | Pr(>\|t\|) |
| (Intercept) | 0.36 | 0.03 | 11.69 | <2e-16 |
|  | edf | Ref.df | F | p-value |
| s(chla.mn) | 5.84E-08 | 9 | 0 | 0.79 |
| s(sst.mn) | 6.19E-08 | 9 | 0 | 0.83 |
| R-sq.(adj) | -9.32E-10 |  |  |  |

**(3) 17:0**

| LME Random effects | SD |  |  |  |
| --- | --- | --- | --- | --- |
| ~\|sampling month | 0.06 |  |  |  |
| Residual | 0.41 |  |  |  |
| AIC | BIC | logLik |  |  |
| 147.59 | 161.89 | -68.80 |  |  |
| GAM | Estimate | Std. Error | t value | Pr(>\|t\|) |
| (Intercept) | 1.36 | 0.05 | 29.92 | <2e-16 |
|  | edf | Ref.df | F | p-value |
| s(chla.mn) | 3.50E-07 | 9 | 0 | 0.55 |
| s(sst.mn) | 5.57E-01 | 9 | 0.14 | 0.14 |
| R-sq.(adj) | 0.0192 |  |  |  |

**(4) 18:0**

| LME Random effects | SD |  |  |  |
| --- | --- | --- | --- | --- |
| ~\|sampling month | 0.85 |  |  |  |
| Residual | 2.07 |  |  |  |
| AIC | BIC | logLik |  |  |
| 572.51 | 586.81 | -281.26 |  |  |
| GAM | Estimate | Std. Error | t value | Pr(>\|t\|) |
| (Intercept) | 6.43 | 0.43 | 15.06 | <2e-16 |
|  | edf | Ref.df | F | p-value |
| s(chla.mn) | 2.43E-04 | 9 | 0 | 0.31 |
| s(sst.mn) | 6.20E-01 | 9 | 0.17 | 0.12 |
| R-sq.(adj) | 0.05 |  |  |  |

**(5) 14:1n5**

| LME Random effects | SD |  |  |  |
| --- | --- | --- | --- | --- |
| ~\|sampling month | 0.12 |  |  |  |
| Residual | 0.30 |  |  |  |
| AIC | BIC | logLik |  |  |
| 74.18 | 88.48 | -32.09 |  |  |
| GAM | Estimate | Std. Error | t value | Pr(>\|t\|) |
| (Intercept) | 0.60 | 0.06 | 9.88 | <2e-16 |
|  | edf | Ref.df | F | p-value |
| s(chla.mn) | 2.92E-08 | 9 | 0 | 0.82 |
| s(sst.mn) | 6.68E-08 | 9 | 0 | 0.82 |
| R-sq.(adj) | -4.32E-10 |  |  |  |

**(6) 16:1n7**

| LME Random effects | SD |  |  |  |
| --- | --- | --- | --- | --- |
| ~\|sampling month | 0.49 |  |  |  |
| Residual | 0.95 |  |  |  |
| AIC | BIC | logLik |  |  |
| 373.04 | 387.34 | -181.52 |  |  |
| GAM | Estimate | Std. Error | t value | Pr(>\|t\|) |
| (Intercept) | 2.49 | 0.24 | 10.47 | <2e-16 |
|  | edf | Ref.df | F | p-value |
| s(chla.mn) | 1.34E-07 | 9 | 0 | 0.43 |
| s(sst.mn) | 5.02E-08 | 9 | 0 | 0.93 |
| R-sq.(adj) | 5.91E-09 |  |  |  |

**(7) 20:1**

| LME Random effects | SD |  |  |  |
| --- | --- | --- | --- | --- |
| ~\|sampling month | 0.61 |  |  |  |
| Residual | 1.90 |  |  |  |
| AIC | BIC | logLik |  |  |
| 547.59 | 561.89 | -268.80 |  |  |
| GAM | Estimate | Std. Error | t value | Pr(>\|t\|) |
| (Intercept) | 5.69 | 0.32 | 17.68 | <2e-16 |
|  | edf | Ref.df | F | p-value |
| s(chla.mn) | 1.69E-06 | 9 | 0 | 0.32 |
| s(sst.mn) | 1.31E-07 | 9 | 0 | 0.62 |
| R-sq.(adj) | 1.40E-08 |  |  |  |

**(8) 22:1n9**

| LME Random effects | SD |  |  |  |
| --- | --- | --- | --- | --- |
| ~\|sampling month | 0.60 |  |  |  |
| Residual | 1.75 |  |  |  |
| AIC | BIC | logLik |  |  |
| 527.77 | 542.06 | -258.88 |  |  |
| GAM | Estimate | Std. Error | t value | Pr(>\|t\|) |
| (Intercept) | 1.78 | 0.31 | 5.71 | 7.67E-08 |
|  | edf | Ref.df | F | p-value |
| s(chla.mn) | 7.72E-08 | 9 | 0 | 0.77 |
| s(sst.mn) | 4.64E-08 | 9 | 0 | 0.87 |
| R-sq.(adj) | -9.38E-10 |  |  |  |

**(9) 24:1n9**

| LME Random effects | SD |  |  |  |
| --- | --- | --- | --- | --- |
| ~\|sampling month | 0.15 |  |  |  |
| Residual | 0.45 |  |  |  |
| AIC | BIC | logLik |  |  |
| 180.58 | 194.88 | -85.29 |  |  |
| GAM | Estimate | Std. Error | t value | Pr(>\|t\|) |
| (Intercept) | 1.92 | 0.08 | 24.30 | <2e-16 |
|  | edf | Ref.df | F | p-value |
| s(chla.mn) | 8.39E-01 | 9 | 0.45 | 0.02 |
| s(sst.mn) | 3.82E-01 | 9 | 0.07 | 0.20 |
| R-sq.(adj) | 0.10 |  |  |  |

**(10) 18:2n6t**

| LME Random effects | SD |  |  |  |
| --- | --- | --- | --- | --- |
| ~\|sampling month | 0.39 |  |  |  |
| Residual | 0.79 |  |  |  |
| AIC | BIC | logLik |  |  |
| 323.51 | 337.81 | -156.75 |  |  |
| GAM | Estimate | Std. Error | t value | Pr(>\|t\|) |
| (Intercept) | 1.31 | 0.19 | 6.94 | 1.72E-10 |
|  | edf | Ref.df | F | p-value |
| s(chla.mn) | 2.53E-08 | 9 | 0 | 0.89 |
| s(sst.mn) | 8.50E-08 | 9 | 0 | 0.72 |
| R-sq.(adj) | 1.40E-09 |  |  |  |

**(11) 18:3n6**

| LME Random effects | SD |  |  |  |
| --- | --- | --- | --- | --- |
| ~\|sampling month | 0.27 |  |  |  |
| Residual | 0.51 |  |  |  |
| AIC | BIC | logLik |  |  |
| 212.58 | 226.88 | -101.29 |  |  |
| GAM | Estimate | Std. Error | t value | Pr(>\|t\|) |
| (Intercept) | 0.82 | 0.13 | 6.26 | 5.48E-09 |
|  | edf | Ref.df | F | p-value |
| s(chla.mn) | 3.43E-08 | 9 | 0 | 0.66 |
| s(sst.mn) | 1.21E-07 | 9 | 0 | 0.53 |
| R-sq.(adj) | 6.38E-09 |  |  |  |

**(12) 20:2**

| LME Random effects | SD |  |  |  |
| --- | --- | --- | --- | --- |
| ~\|sampling month | 0.23 |  |  |  |
| Residual | 0.34 |  |  |  |
| AIC | BIC | logLik |  |  |
| 107.61 | 121.91 | -48.81 |  |  |
| GAM | Estimate | Std. Error | t value | Pr(>\|t\|) |
| (Intercept) | 1.36 | 0.11 | 12.59 | <2e-16 |
|  | edf | Ref.df | F | p-value |
| s(chla.mn) | 5.08E-08 | 9 | 0 | 0.71 |
| s(sst.mn) | 3.85E-08 | 9 | 0 | 0.73 |
| R-sq.(adj) | 2.64E-09 |  |  |  |

**(13) 20:3n6**

| LME Random effects | SD |  |  |  |
| --- | --- | --- | --- | --- |
| ~\|sampling month | 0.25 |  |  |  |
| Residual | 0.44 |  |  |  |
| AIC | BIC | logLik |  |  |
| 174.75 | 189.05 | -82.37 |  |  |
| GAM | Estimate | Std. Error | t value | Pr(>\|t\|) |
| (Intercept) | 0.81 | 0.12 | 6.89 | 2.29E-10 |
|  | edf | Ref.df | F | p-value |
| s(chla.mn) | 2.20E-08 | 9 | 0 | 0.57 |
| s(sst.mn) | 2.03E-07 | 9 | 0 | 0.38 |
| R-sq.(adj) | 1.66E-08 |  |  |  |

**(14) 20:4n6**

| LME Random effects | SD |  |  |  |
| --- | --- | --- | --- | --- |
| ~\|sampling month | 0.43 |  |  |  |
| Residual | 1.27 |  |  |  |
| AIC | BIC | logLik |  |  |
| 447.25 | 461.55 | -218.62 |  |  |
| GAM | Estimate | Std. Error | t value | Pr(>\|t\|) |
| (Intercept) | 2.50 | 0.22 | 11.14 | <2e-16 |
|  | edf | Ref.df | F | p-value |
| s(chla.mn) | 9.51E-01 | 9 | 0.95 | 0.0027 |
| s(sst.mn) | 1.36E-07 | 9 | 0 | 0.70 |
| R-sq.(adj) | 0.0793 |  |  |  |

**(15) 22:2n6**

| LME Random effects | SD |  |  |  |
| --- | --- | --- | --- | --- |
| ~\|sampling month | 0.34 |  |  |  |
| Residual | 0.60 |  |  |  |
| AIC | BIC | logLik |  |  |
| 255.71 | 270.01 | -122.86 |  |  |
| GAM | Estimate | Std. Error | t value | Pr(>\|t\|) |
| (Intercept) | 0.94 | 0.16 | 5.79 | 5.22E-08 |
|  | edf | Ref.df | F | p-value |
| s(chla.mn) | 2.71E-08 | 9 | 0 | 0.65 |
| s(sst.mn) | 1.10E-07 | 9 | 0 | 0.53 |
| R-sq.(adj) | 6.07E-09 |  |  |  |

**(16) 20:5n3**

| LME Random effects | SD |  |  |  |
| --- | --- | --- | --- | --- |
| ~\|sampling month | 0.53 |  |  |  |
| Residual | 1.47 |  |  |  |
| AIC | BIC | logLik |  |  |
| 483.52 | 497.82 | -236.76 |  |  |
| GAM | Estimate | Std. Error | t value | Pr(>\|t\|) |
| (Intercept) | 6.10 | 0.27 | 22.32 | <2e-16 |
|  | edf | Ref.df | F | p-value |
| s(chla.mn) | 7.20E-08 | 9 | 0 | 0.66 |
| s(sst.mn) | 4.30E-08 | 9 | 0 | 0.76 |
| R-sq.(adj) | -1.14E-09 |  |  |  |
