# Supplementary material for: Top predator reveals the stability of prey community in the western subarctic Pacific
Source: PLoS One. 2020 Jun 19;15(6):e0234905. doi: 10.1371/journal.pone.0234905 (PMC7304915; doi:10.1371/journal.pone.0234905)
Supplement: Attachment — Submitted filename: Lin and Chen Lamb reviews.pdf [file pone.0234905.s003.pdf]

Lin and Chen use isotopic ratios from top predators to evaluate the stability of prey communities in the sub-Arctic ocean.

General Comments

This study is interesting, rooted in strong theory and logical predictions, and in an interesting and difficult to access part of the world. However, the writing style and readability of the paper are deficient, I wonder if this has been critically reviewed by a native English speaker? If not that would be a good first step of a thorough rewrite. As it stands, the MS is difficult to read due to widespread grammatical and spelling errors, often hindering appreciation of the science being reported. I note several instances in my specific comments, but the authors should review the manuscript thoroughly with a native English speaker.

The abstract lacks critical background information: over what time period and at what frequency were biological and environmental measurements made? To what degree did SST and chlorophyll vary over the course of the study, enough to make us expect that diets should be changing as well?

To what extent do the months sampled create bias in the findings of the paper? I would guess that July – November is the warmest and perhaps most stable period of ocean temperatures in the region. Might the prey community and isotopic values change more during winter months?

Specific Comments

Line 18, 21, and elsewhere: prey community (remove -)

Line 20: squid *Ommastrephes bartramii*, an active top marine predator, as a biological sampler to investigate the…

Line 23 and throughout the manuscript: Entire MS needs thorough revision by a native English speaker, for instance this sentence reads much better as "No significant differences in stable isotope ratios were detected among sampling months."

Please state the period of time over which sampling occurred

Line 38: stability and subsequent service of ecosystem**s** [3-4]. As stability is ~~the~~ central to an ecosystem

Here and throughout the manuscript are many out of place "the", mistakes with plurals, etc.

Line 72: Alternative to what? Please give examples and how they compare

Line 74: Exemplary run-on sentence without clear structure, requiring substantial re-write by native English speaker.

Line 93: Apart from what? Do you mean trophic level in width? The cited study found that *O. bartramii* is substantially more than a trophic level apart from another species of squid, *S. oualaniensis*, which you do not bring up here.

Line 95 and Line 340-343: This is the one critical concern I have about the manuscript regarding methodology and assumptions, does a change in diet necessarily indicate prey stability? Could the squid change its feeding behavior, location, or timing to keep up with changes in prey distributions? For instance, their daily vertical migrations integrate across several ocean ecosystems, and prey could become scarce at one depth and abundant at another? Are you assuming that predation rate and prey abundance are density-dependent?

Line 105: still exi**s**t

Line 113: Owing to closing? This is not clear

Line 182: Here or in results for this method, indicate what a normal non-overlapping SEAc proportion looks like, what do high or low values tell you? Orientate your reader to the method

Line 234: evidenced by the (**large?)** non-overlap (SEAc) proportion (Table 2) and the **high degree of overlap for** ellipse**s** **of different months**

Line 246: No significant difference in total fatty acid values was found among sample months (ANOVA…

Line 381: This is a nice theoretical paragraph. Please include some discussion of 1) the implications of the open ocean environment (homogeneous, large, low and unpredictable encounter rates with other organisms, etc.) for the use of stable isotopes for inference of prey dynamics, would this method be applicable in other systems? And 2) the implications of this ecosystem being based on micro-zooplankton instead of phytoplankton, as mentioned on Line 116, for community stability. Where do these micro-zooplankton come from, and are they inherently more stable than a phytoplankton-based food web?
