# Supplementary material for: Top predator reveals the stability of prey community in the western subarctic Pacific
Source: PLoS One. 2020 Jun 19;15(6):e0234905. doi: 10.1371/journal.pone.0234905 (PMC7304915; doi:10.1371/journal.pone.0234905)
Supplement: Attachment — Submitted filename: Response to Reviewers_R1.docx [file pone.0234905.s004.docx]

PONE-D-19-29709
Top predator reveals the stability of prey-community in the western subarctic Pacific
PLOS ONE

Reviewer #1: Lin and Chen use isotopic ratios from top predators to evaluate the stability of prey communities in the sub-Arctic ocean.

**General Comments**

*This study is interesting, rooted in strong theory and logical predictions, and in an interesting and difficult to access part of the world. However, the writing style and readability of the paper are deficient, I wonder if this has been critically reviewed by a native English speaker? If not that would be a good first step of a thorough rewrite. As it stands, the MS is difficult to read due to widespread grammatical and spelling errors, often hindering appreciation of the science being reported. I note several instances in my specific comments, but the authors should review the manuscript thoroughly with a native English speaker.*

***Response:*** Thank you so much for your recognition of our work. We asked two English-speaking colleagues (based in Australia and the US) to review the revised manuscript and they provided extensive suggestions to improve readability, grammar, and spelling. We trust that the paper is now of the standard expected of the journal.

*The abstract lacks critical background information: over what time period and at what frequency were biological and environmental measurements made? To what degree did SST and chlorophyll vary over the course of the study, enough to make us expect that diets should be changing as well?*

***Response:*** We have added information about the sampling period as well as the SST and Chl-a data. The samples were collected monthly from July to November 2016 while the SST and Chl-a data were downloaded from the NOAA ERDDAP (Version 1.82), at a monthly mean scale for each sampling station. SST varied from 13.44±0.74 ℃ to 19.83±0.22 ℃, and Chl-a varied from 0.25±0.02 mg m^-3^ to 0.61±0.07 mg m^-3^. The statistical analysis indicated that both SST and Chl-a differed significantly among sampling months. Details please see our revised MS.

*To what extent do the months sampled create bias in the findings of the paper? I would guess that July – November is the warmest and perhaps most stable period of ocean temperatures in the region. Might the prey community and isotopic values change more during winter months?*

***Response:*** Based on the SST and Chl-*a* data from the NOAA ERDDAP, we know that both environmental variables vary significantly during our survey period (July to November). Monthly mean SST varied from 13.44℃ to 19.83℃, and monthly mean Chl-a varied from 0.25 mg m^-3^ to 0.61 mg m^-3^.

Our data only cover summer and autumn, and the findings might be not applicable to other seasons such as winter when productivity is usually low and spring when primary production is the highest in the Oyashio area in the northwest Pacific. However, the life history strategy of some copepods in the region may be independent of the environment (temperature, presence or absence of the spring bloom and the timing difference of primary production) [1]. Based on your suggestions, we have included some discussion about this issue in the final paragraph of the Discussion section. For details please see our revised MS.

**Specific Comments**

*Line 18, 21, and elsewhere: prey community (remove -)*

***Response:*** We have remove the hyphen here and in other places in the MS.

*Line 20: squid Ommastrephes bartramii, an active top marine predator, as a biological sampler to investigate the…*

***Response:*** We have revised the text following your suggestion.

*Line 23 and throughout the manuscript: Entire MS needs thorough revision by a native English speaker, for instance this sentence reads much better as “No significant differences in stable isotope ratios were detected among sampling months.”*

***Response:*** We have rephrased it, and asked a native English speaker to edit our manuscript.

*Please state the period of time over which sampling occurred*

***Response:*** We have revised it as “Squid were collected monthly from July to November 2016. There were no significant differences among months in stable isotopes (δ^13^C and δ^15^N) in the digestive gland, a fast turnover organ reflecting recent dietary information.”

*Line 38: stability and subsequent service of ecosystems [3-4]. As stability is the central to an ecosystem*

***Response:*** We have rephrased it.

*Here and throughout the manuscript are many out of place “the”, mistakes with plurals, etc.*

***Response:*** These types of errors have been corrected in the revised version of the manuscript.

*Line 72: Alternative to what? Please give examples and how they compare*

***Response:*** Sorry for this lack of clarity. Based on your suggestion, we have revised the text to “Biochemical tracers are considered to be a complementary or even alternative and cost-effective tool to stomach content analysis for examining major changes in trophic structure and ecosystem productivity.” Also, we have outlined one example that used fatty acid profiles to reveal temporal dietary shifts in *Todarodes filippovae* related to site-specific oceanography and ecosystem structure in the continental slope waters in the Southern Ocean. For details please see our revised MS.

*Line 74: Exemplary run-on sentence without clear structure, requiring substantial re-write by native English speaker.*

***Response:*** We have revised this sentence and other sentences that seem to have similar problems.

*Line 93: Apart from what? Do you mean trophic level in width? The cited study found that O. bartramii is substantially more than a trophic level apart from another species of squid, S. oualaniensis, which you do not bring up here.*

***Response:*** Yes, you are right. However, we have revised this paragraph following your suggestions below, and this text has been removed. For details, please see our revised MS.

*Line 95 and Line 340-343: This is the one critical concern I have about the manuscript regarding methodology and assumptions, does a change in diet necessarily indicate prey stability? Could the squid change its feeding behavior, location, or timing to keep up with changes in prey distributions? For instance, their daily vertical migrations integrate across several ocean ecosystems, and prey could become scarce at one depth and abundant at another? Are you assuming that predation rate and prey abundance are density-dependent?*

**Response:** Thanks for your insightful comments and suggestions. We have added the following text to address this key issue: “More importantly, *O. bartramii* is a high trophic level species, with an average δ^15^N value up to 13.6‰ [2-5], which occupies a similar trophic position as other top predators such as albatrosses (mean δ^15^N, 12.0‰ for *Diomedea immutabilis*; 14.4‰ for *Diomedea nigripes*) [2], and sharks (*Prionace glauca*, mean 12.1‰) [6]. *O. bartramii* is an opportunistic generalist that preys on a wide variety of species, including crustaceans, fishes and cephalopods [3, 7-9]. The diet of *O. bartramii* varies spatial-temporally given the associated prey community, e.g., it may feed on transitional-water species during its northward feeding migration [8], migratory mesopelagic species in the epipelagic zone at night [10] and non-migratory species during the day in the mesopelagic zone [8]. *O. bartramii* therefore has the potential to be an ideal trophic indicator of ecosystem functioning [11], and represents a way integrate the ecological dynamics [12].”

We agree. This species shifts diet with ontogeny, possibly driven by changes in body size and spatial-temporal distribution [9]. However, feeding behavior appears to be associated with prey composition. For instance, *O. bartramii* in the central North Pacific appears to mainly feed on transitional-water species such as *Symbolophorus californiensis* and *Onychoteuthis borealijaponica* during northward feeding migrations from the transitional zone to the transitional domain from May to July, since the seasonal migration patterns of prey and predators are similar spatial-temporally [8]. Additionally, although this species has been recognized as an epipelagic feeder and feeds at night when the bulk of the mesopelagic prey species migrate into the epipelagic layers [10], squids of larger size also feed on non-migratory species such as *Protomyctophum thompsoni* and *Bathylagus ochotensis* during the day in the mesopelagic zone where they vertically migrate to [8].

Therefore, *O. bartramii* has the potential to be a trophic indicator of the dynamics of prey communities within an ecosystem. Also, due to their abundance, *O. bartramii* has a major impact on the structure of the food web via top-down effects on prey [5, 13]. Hence, to a lesser extent, studies on the dynamics of species preyed on by *O. bartramii* can determine the nature of the ecosystem they encounter.

Based on your suggestions and concerns, we have carefully revised these sentences. Hopefully the modifications and descriptions above address your concerns.

*Line 105: still exist*

***Response:*** We have cut down the sentence, so this term has been deleted.

*Line 113: Owing to closing? This is not clear*

**Response:** Corrected. We have deleted the word “closing”.

*Line 182: Here or in results for this method, indicate what a normal non-overlapping SEAc proportion looks like, what do high or low values tell you? Orientate your reader to the method*

**Response:** Sorry. We have miswritten this sentence, and we have revised it to: “the overlap as the proportion of the sum of the non-overlapping ellipse areas (non-overlap SEAc proportion) based on 1,000 replications”. We have also included some text about the meaning of non-overlapping SEAc, which ranges from 0 when there is no overlap in isotopic niche widths among groups to 1 when there is complete overlap in the isotopic niche widths between groups.

*Line 234: evidenced by the (large?) non-overlap (SEAc) proportion (Table 2) and the high degree of overlap for ellipses of different months*

**Response:** Correct, it is the large non-overlap (SEAc) proportion. Accordingly, we have followed your suggestion and revised it to “confirmed by the high non-overlap (SEAc) proportions (Table 2) and the considerable overlap of the ellipses for the different months (Fig 2 B)”.

*Line 246: No significant difference in total fatty acid values was found among sample months (ANOVA…*

**Response:** Agreed, we have revised it.

*Line 381: This is a nice theoretical paragraph. Please include some discussion of 1) the implications of the open ocean environment (homogeneous, large, low and unpredictable encounter rates with other organisms, etc.) for the use of stable isotopes for inference of prey dynamics, would this method be applicable in other systems? And 2) the implications of this ecosystem being based on micro-zooplankton instead of phytoplankton, as mentioned on Line 116, for community stability. Where do these micro-zooplankton come from, and are they inherently more stable than a phytoplankton-based food web?*

**Response:** Based on your suggestions, we have included some discussion about the zooplankton-based food web in the last paragraph of the Discussion in the revised MS. In the Gyre, the zooplankton assemblage is dominated by large interzonal copepods [1]. The zooplankton feeds and grows at the surface layer for a few months, and resides at depth for remaining months, with 1 or 2 years life cycles [1]. Meanwhile, many mesozooplankton including copepods, euphausiids and salps show intensive patchiness in their spatial distributions, which create local zones of high prey availability for predators [14]. However, it is not possible to know whether a zooplankton-based food web is more stable than a phytoplankton-based food web here. More work is needed to address the hypothesis. Also, we have divided this pargragph into three as it is too large.

Yes, you are right, and the methodology of stable isotopes can be used to infer prey dynamics in other systems. This is because stable nitrogen isotope ratios (δ15N) can be used to identify trophic positions with enrichment of about 3‰ per trophic level [15, 16]. On the other hand, the stable carbon isotope ratio (δ13C) is effective for determining dietary sources, because the ratios in food sources and consumers in higher trophic levels are similar [15, 16]. Regarding fatty acids, many of them can only be biosynthesized by certain phytoplankton and macroalgae species and become essential dietary components to higher trophic levels, where the fatty acids are assimilated without, or with only minimal, modification [17]. Therefore, it is possible to investigate the prey dynamics of a predator by combining these methods.

We have not included text about using stable isotopes to infer prey dynamics for this open ocean system. This is because this paragraph is primarily to illustrate possible reasons why the prey community is stable under a variable environment. Hopefully you agree with us.

*While revising your submission, please upload your figure files to the Preflight Analysis and Conversion Engine (PACE) digital diagnostic tool, <https://pacev2.apexcovantage.com/>. PACE helps ensure that figures meet PLOS requirements. To use PACE, you must first register as a user. Registration is free. Then, login and navigate to the UPLOAD tab, where you will find detailed instructions on how to use the tool. If you encounter any issues or have any questions when using PACE, please email us at* [*figures@plos.org*](mailto:figures@plos.org)*. Please note that Supporting Information files do not need this step.*

**Response:** We have uploaded the figure files to PACE, which indicates that all the figures meet PLOS requirements.

**Reference**

1.	Tsuda A, Saito H, Kasai H. Life histories of Eucalanus bungii and Neocalanus cristatus (Copepoda: Calanoida) in the western subarctic Pacific Ocean. Fish Oceanogr. 2004;13(s1):10-20. doi: 10.1111/j.1365-2419.2004.00315.x.

2.	Gould P, Ostrom P, Walker W. Trophic relationships of albatrosses associated with squid and large-mesh drift-net fisheries in the North Pacific Ocean. Canadian Journal of Zoology. 1997;75(4):549-62. doi: 10.1139/z97-068.

3.	Parry MP. The trophic ecology of two ommastrephid squid species, *Ommastrephes bartramii* and *Sthenoteuthis oualaniensis*, in the north Pacific sub-tropical gyre. Manoa: University of Hawaii; 2003.

4.	Parry M. Trophic variation with length in two ommastrephid squids, Ommastrephes bartramii and Sthenoteuthis oualaniensis. Mar Biol. 2008;153(3):249-56. doi: 10.1007/s00227-007-0800-3.

5.	Kato Y, Sakai M, Nishikawa H, Igarashi H, Ishikawa Y, Vijai D, et al. Stable isotope analysis of the gladius to investigate migration and trophic patterns of the neon flying squid (*Ommastrephes bartramii*). Fish Res. 2016;173:169-74. doi: <https://doi.org/10.1016/j.fishres.2015.09.016>.

6.	Fujinami Y, Nakatsuka S, Ohshimo S. Feeding Habits of the Blue Shark (*Prionace glauca*) in the Northwestern Pacific Based on Stomach Contents and Stable Isotope Ratios. Pacific Science. 2018;72(1):21-39. doi: 10.2984/72.1.2.

7.	Matthew P. Feeding behavior of two ommastrephid squids *Ommastrephes bartramii* and *Sthenoteuthis oualaniensis* off Hawaii. Mar Ecol Prog Ser. 2006;318:229-35. doi: 10.3354/meps318229.

8.	Watanabe H, Kubodera T, Ichii T, Kawahara S. Feeding habits of neon flying squid *Ommastrephes bartramii* in the transitional region of the central North Pacific. Mar Ecol Prog Ser. 2004;266:173-84. doi: 10.3354/meps266173.

9.	Jereb P, Roper CFE. Cephalopods of the world. An annotated and illustrated catalogue of cephalopod species known to date. Volume 2. Myopsid and Oegopsid Squids. Rome: FAO; 2010. 605 p.

10.	Lipiński MR, Linkowski TB. Food of the squid Ommastrephes bartramii (Lesueur, 1821) from the South-West Atlantic Ocean. S Afr J Mar Sci. 1988;6(1):43-6. doi: 10.2989/025776188784480573.

11.	Boyle P, Rodhouse P. Cephalopods: ecology and fisheries. Oxford, UK: Wiley-Blackwell; 2005. 464 p.

12.	Bell G, Fortier-Dubois É. Trophic dynamics of a simple model ecosystem. Proceedings of the Royal Society B: Biological Sciences. 2017;284(1862):20171463. doi: 10.1098/rspb.2017.1463.

13.	Ichii T, Mahapatra K, Sakai M, Wakabayashi T, Okamura H, Igarashi H, et al. Changes in abundance of the neon flying squid *Ommastrephes bartramii* in relation to climate change in the central North Pacific Ocean. Mar Ecol Prog Ser. 2011;441:151-64.

14.	Mackas DL, Tsuda A. Mesozooplankton in the eastern and western subarctic Pacific: community structure, seasonal life histories, and interannual variability. Progress in Oceanography. 1999;43(2):335-63. doi: <https://doi.org/10.1016/S0079-6611(99)00012-9>.

15.	Post DM. Using stable isotopes to estimate trophic position: models, methods, and assumptions. Ecology. 2002;83(3):703-18. doi: 10.1890/0012-9658(2002)083[0703:USITET]2.0.CO;2.

16.	Layman CA, Araujo MS, Boucek R, Hammerschlag-Peyer CM, Harrison E, Jud ZR, et al. Applying stable isotopes to examine food-web structure: an overview of analytical tools. Biol Rev. 2012;87(3):545-62. doi: 10.1111/j.1469-185X.2011.00208.x.

17.	Iverson SJ. Tracing aquatic food webs using fatty acids: from qualitative indicators to quantitative determination. In: Kainz M, Brett MT, Arts MT, editors. Lipids in Aquatic Ecosystems. New York, NY: Springer New York; 2009. p. 281-308.
