# Supplementary material for: Top predator reveals the stability of prey community in the western subarctic Pacific
Source: PLoS One. 2020 Jun 19;15(6):e0234905. doi: 10.1371/journal.pone.0234905 (PMC7304915; doi:10.1371/journal.pone.0234905)
Supplement: Attachment — Submitted filename: Second review for Top Predator Plos one.docx [file pone.0234905.s005.docx]

Second review for “Top predator reveals the stability of prey community in the western subarctic Pacific”

**General Comments**

Lin and Chen have submitted a considerably improved version of their manuscript. I find very few faults in the English writing (but see a few in specific comments). The manuscript is interesting and thorough and is near ready for publication. I think there could still be a bit more exploration of the limitations of the data and possible interpretations of the surprisingly stable dietary dynamics despite changes in oceanographic parameters. Some further development considering what the squid are actually eating, what features of their ecology promote this apparent ecological stability, how you might see different trends in different predator species or across a larger coverage of annual trends, etc. See below for some suggestions for expanding the discussion.

**Specific Comments**

Line 18: The stability of ~~the~~ ecosystems; Also this sentence might be better finished: “but changes in the composition and abundance of prey species are poorly understood, especially in open ocean ecosystems.”

Line 38-41: These two sentences are uninformative, and the use of the word “prey” is misleading (almost every animal is prey to some other animal). Suggest re-writing perhaps as “Concern about ecosystem functioning highlights the need for a better understanding of how the composition and abundance of species in natural communities respond to environmental change. For instance, predatory animals are susceptible to reduction or extirpation of available prey due to environmental processes, which undermines the stability of ecosystems and the services they provide.”

Line 54: Squids grow **quickly** (or rapidly)

Line 64: such as stable isotope**s**

Line 66: dietary history over **a range of temporal scales** (since this is more appropriate to the use of different tissues that vary in their turnover rates)

Line 73: match ~~of~~ those of their prey

Line 78: with little or **no** modification

Line 84: as **a** biological sampler..

Line 100: suggest: … and represents a way of integrating ecological dynamics over a large area and across several ecosystems that are difficult to study directly.

Line 100: isotope ratios and fatty acids (no comma)

Line 106: suggest: … quantifying community dynamics in response to environmental change.

Line 125 (and elsewhere) indent on first sentence of each paragraph

Line 127: considered to **be** one of active…

Line 219: Since tables and figures are often seen separated from the main text, consider defining SEAc in the caption

Line 263: What is the relationship between the temperature and Chl a variation observed in the study months in comparison to the total variation observed throughout the year? If you included a wider range of the possible environmental variation and sampling months would you expect to see a different result?

Line 304: There is some room for discussion I think of what the squid are actually eating, perhaps through comparison with other studies that have gut content analysis paired with stable isotope or fatty acid analysis.

Line 325: If they are feeding on a wide range of diet items, would you not expect there to be considerable variation among squid samples in their diet, whether among individuals within a season or between seasons? You could test for differences in multivariate dispersion in the fatty acid nMDS profiles using betadisper() in the vegan package to examine whether variation in diet among individuals varies over time.

Line 352: Indeed this was a finding I did not expect when reading the intro and methods. More could be made of this surprising finding. I imagine much of the stability over time in diet reflects the large area and multiple ecosystems (depths) across which squid integrate their diets. Would you expect different results if you looked at more vertically restricted species? Or more specialist predators?

Line 357: evolutiona**rily** flexible

Line 367: copepods predominate~~s~~ the

Line 372: long time period (no -)

Line 380: part of **the** Western…
