# Supplementary material for: Top predator reveals the stability of prey community in the western subarctic Pacific
Source: PLoS One. 2020 Jun 19;15(6):e0234905. doi: 10.1371/journal.pone.0234905 (PMC7304915; doi:10.1371/journal.pone.0234905)
Supplement: Attachment — Submitted filename: Response to Reviewers_R2.docx [file pone.0234905.s006.docx]

PONE-D-19-29709R1
Top predator reveals the stability of prey community in the western subarctic Pacific
PLOS ONE

Reviewer #1: Second review for “Top predator reveals the stability of prey community in the western subarctic Pacific”

**General Comments**

*Lin and Chen have submitted a considerably improved version of their manuscript. I find very few faults in the English writing (but see a few in specific comments). The manuscript is interesting and thorough and is near ready for publication. I think there could still be a bit more exploration of the limitations of the data and possible interpretations of the surprisingly stable dietary dynamics despite changes in oceanographic parameters. Some further development considering what the squid are actually eating, what features of their ecology promote this apparent ecological stability, how you might see different trends in different predator species or across a larger coverage of annual trends, etc. See below for some suggestions for expanding the discussion.*

***Response***: Thank you so much for your insightful comments. We have revised the MS based on your constructive suggestions. Details please see our revised version.

**Specific Comments**

*Line 18: The stability of the ecosystems; Also this sentence might be better finished: “but changes in the composition and abundance of prey species are poorly understood, especially in open ocean ecosystems.”*

***Response***: We have revised this sentence following your suggestion.

*Line 38-41: These two sentences are uninformative, and the use of the word “prey” is misleading (almost every animal is prey to some other animal). Suggest re-writing perhaps as “Concern about ecosystem functioning highlights the need for a better understanding of how the composition and abundance of species in natural communities respond to environmental change. For instance, predatory animals are susceptible to reduction or extirpation of available prey due to environmental processes, which undermines the stability of ecosystems and the services they provide.”*

***Response***: We have revised the sentences following your suggestions.

*Line 54: Squids grow quickly (or rapidly)*

***Response***: We have revised it as “Squids grow rapidly”.

*Line 64: such as stable isotopes*

***Response***: We have revised it as “stable isotopes”.

*Line 66: dietary history over a range of temporal scales (since this is more appropriate to the use of different tissues that vary in their turnover rates)*

***Response***: We have revised it as “a range of temporal scales”.

*Line 73: match of those of their prey*

***Response***: We have revised it as “match of those of their prey”.

*Line 78: with little or no modification*

***Response***: We have revised it as “with little or no modification”.

*Line 84: as a biological sampler..*

***Response***: We have revised it as “as a biological sampler”.

*Line 100: suggest: … and represents a way of integrating ecological dynamics over a large area and across several ecosystems that are difficult to study directly.*

***Response***: We have revised it as “… and represents a way of integrating ecological dynamics over a large area and across several ecosystems that are difficult to study directly”. Thanks!

*Line 100: isotope ratios and fatty acids (no comma)*

***Response***: We have deleted the comma.

*Line 106: suggest: … quantifying community dynamics in response to environmental change.*

***Response***: We have revised it as “… quantifying community dynamics in response to environmental change”.

*Line 125 (and elsewhere) indent on first sentence of each paragraph*

***Response***: We have indented the first sentence of each paragraph throughout the MS.

*Line 127: considered to be one of active…*

***Response***: We have revised it.

*Line 219: Since tables and figures are often seen separated from the main text, consider defining SEAc in the caption*

***Response***: We have defined SEAc and non-overlap SEAc proportion in the table caption.

*Line 263: What is the relationship between the temperature and Chl a variation observed in the study months in comparison to the total variation observed throughout the year? If you included a wider range of the possible environmental variation and sampling months would you expect to see a different result?*

***Response:*** Accordingly, we have analyzed the relationship between SST and Chl-*a* among the study months, and the results showed that there is a light correlation between SST and Chl-*a* (*R_adi._^2^*=0.32), though it is significant (F=-3.45, P=0.0023). We have also checked the collinearity between these two predictors, and the results indicated that the collinearity is relatively low (VIF=1.54). So, it is suitable to use these two factors simultaneously to predict the potential effects of the ambient environment on the dynamics of the prey community here.

Regarding SST and Chl-*a* throughout the sampling year, we have analyzed their variation by month within a rectangle region (153°-160°E, 42°-46°N) including all of the sampling stations in the gyre (**Figure 1 below**). We can find that both SST and Chl-*a* of the sampling months have a similar change pattern compared to that of the same months of the sampling year. We can also find that although sea surface temperature was the warmest during the sampling months in the region, the primary production (indication by Cha-a) exhibits a very similar variation to that during the first half of the year. It seems like that the primary production in this region is independent of sea surface temperature, possibly due to the nutrient rich [1-3] and the zooplankton-based food web [4, 5] as discussed in the Discussion section.

Because we have no samples collected from other months of the year (according to the fishery company, there are no fisheries in this region in first half of the year as the harsh condition), we are unable to assure to expect a different result. However, due to the similar change of primary production (indication through Chl-a) for the first and second half of the year, it would be possible to expect that the trophic community could be not too much different from our findings here when the analysis was performed for the whole year.

Due to lack of sampling data of the first half year, we did not revise much more in this paragraphs. But as your concerns, we have added the result of collinearity in the last paragraph of “Statistical analysis”. Hope you agree with us in this respect.

Figure 1 The distribution of mean SST and mean Chl-a by month of the sampling year. Data are presented as mean±SD.

*Line 304: There is some room for discussion I think of what the squid are actually eating, perhaps through comparison with other studies that have gut content analysis paired with stable isotope or fatty acid analysis.*

***Response***: Based on your suggestion, we have revised this paragraph by adding some discussions to justify our finding. We are sorry for that we can’t find any references about the feeding habits of *O. bartramii* in the Southwestern Subarctic Gyre. However, there are some of such studies from other regions such as the Central North Pacific and the Kuroshio-Oyashio transition of the western North Pacific. So, we cited them to discuss our findings here.

Based on the previous studies using stomach contents and stable isotopes (unfortunately, no studies using fatty acids yet), we know that the stomach contents of *Ommastrephes bartramii* can effectively reflect the prey items within a given region, and the δ^15^N values in the mantle muscle can indicate the variations of the prey community. So, the stability of δ^15^N and the similarity of niche widths over sampling months would expect that the prey community occupies a similar niche space and does not change in the southwestern part of the gyre. Details please see our revised MS.

*Line 325: If they are feeding on a wide range of diet items, would you not expect there to be considerable variation among squid samples in their diet, whether among individuals within a season or between seasons? You could test for differences in multivariate dispersion in the fatty acid nMDS profiles using betadisper() in the vegan package to examine whether variation in diet among individuals varies over time.*

***Response***: Yes, you are right. Indeed, we have found that nearly half of the individual fatty acids varied significantly between sampling months. Based on your suggestions, we have used betadisper() to test the dispersion of fatty acids among the sampling months, and found that the variance of the fatty acids was significant between sampling months (*F*=6.38, *P*=0.0001048). Considering the obvious overlap (nMDS scaterplots) and high similarity (ANOSIM R values) of the fatty acids among the sampling months, it is rational to conclude that the prey community in the Western Subarctic Gyre is a dynamic equilibrium due to the importance of variations among species for a given ecosystem stability [6]. Therefore, we have added content of “Coupling with the obvious overlap and similarity of the fatty acid compositions (Fig. 3; Table 4),” before the sentence of “Such findings highlight that the prey community in the Western Subarctic Gyre is likely to be in dynamic equilibrium.” Hope you agree with us in this respect.

*Line 352: Indeed this was a finding I did not expect when reading the intro and methods. More could be made of this surprising finding. I imagine much of the stability over time in diet reflects the large area and multiple ecosystems (depths) across which squid integrate their diets. Would you expect different results if you looked at more vertically restricted species? Or more specialist predators?*

***Response***: Sorry for such confusion. In fact, this paragraph is aimed to explain why our findings contradict the general arguments that populations or species may differ in their life-history traits and subsequent biomass as the changes of water temperature and primary productivity. As mentioned in the “response to reviewers” for the first revision (R1), we have divided the original paragraph including this paragraph into three small paragraphs as it is too large. As your concern, we have merged this paragraph with the previous paragraph at this version.

Yes, you are right. The stability of the prey community reflects the large area and multiple ecosystems across which the squid forages. As you mentioned, it would be expected different results if the biological sampler is a specialist predator or lives in a restricted depth range. However, as indicated in the Introduction, *Ommastrephes bartramii* is characterized by the high trophic level in the food web, opportunistic generalist, spatial-temporally shift diets associated with the prey species community, etc. Thus, based on the results found in this work, we are confident that the trophic community is stable within the ecosystem in the southwestern part of the Western Subarctic Gyre in the northwestern Pacific Ocean.

*Line 357: evolutionarily flexible*

***Response***: We have revised it.

*Line 367: copepods predominates the*

***Response***: We have revised it.

*Line 372: long time period (no -)*

***Response***: We have revised it.

*Line 380: part of the Western…*

***Response***: We have revised it.

*While revising your submission, please upload your figure files to the Preflight Analysis and Conversion Engine (PACE) digital diagnostic tool,* [*https://pacev2.apexcovantage.com/*](https://pacev2.apexcovantage.com/)*. PACE helps ensure that figures meet PLOS requirements. To use PACE, you must first register as a user. Registration is free. Then, login and navigate to the UPLOAD tab, where you will find detailed instructions on how to use the tool. If you encounter any issues or have any questions when using PACE, please email us at* [*figures@plos.org*](mailto:figures@plos.org)*. Please note that Supporting Information files do not need this step.*

**Response:** We have uploaded the figure files to PACE, which indicates that all the figures meet PLOS requirements.

References

1.	Yuan X, Talley LD. The subarctic frontal zone in the North Pacific: Characteristics of frontal structure from climatological data and synoptic surveys. Journal of Geophysical Research: Oceans. 1996;101(C7):16491-508. doi: 10.1029/96jc01249.

2.	Qiu B. Kuroshio and Oyashio Currents. In: Steele JH, editor. Encyclopedia of Ocean Sciences. Oxford: Academic Press; 2001. p. 1413-25.

3.	Imai K, Nojiri Y, Tsurushima N, Saino T. Time series of seasonal variation of primary productivity at station KNOT (44°N, 155°E) in the sub-arctic western North Pacific. Deep-Sea Research II. 2002;49(24):5395-408. doi: <https://doi.org/10.1016/S0967-0645(02)00198-4>.

4.	Mackas DL, Tsuda A. Mesozooplankton in the eastern and western subarctic Pacific: community structure, seasonal life histories, and interannual variability. Progress in Oceanography. 1999;43(2):335-63. doi: <https://doi.org/10.1016/S0079-6611(99)00012-9>.

5.	Tsuda A, Saito H, Kasai H. Life histories of *Eucalanus bungii* and *Neocalanus cristatus* (Copepoda: Calanoida) in the western subarctic Pacific Ocean. Fish Oceanogr. 2004;13(s1):10-20. doi: 10.1111/j.1365-2419.2004.00315.x.

6.	McCann KS. The diversity–stability debate. Nature. 2000;405(6783):228-33. doi: 10.1038/35012234.
